# Decoupling Frequencies, Amplitudes and Phases in Nonlinear Optics

**DOI:** 10.1038/s41598-017-07510-3

**Published:** 2017-08-11

**Authors:** Bruno E. Schmidt, Philippe Lassonde, Guilmot Ernotte, Matteo Clerici, Roberto Morandotti, Heide Ibrahim, François Légaré

**Affiliations:** 1 few-cycle Inc., 2890 Rue de Beaurivage, Montreal, H1L 5W5 Qc Canada; 2 INRS-EMT, 1650 Blvd. Lionel Boulet, Varennes, J3X1S2 Qc Canada; 3 University of Glasgow, School of Engineering, G12 8QQ Glasgow, UK; 4 Institute of Fundamental and Frontier Sciences, University of Electronic Science and Technology of China, Chengdu, 610054 China; 5 National Research University of Information Technologies, Mechanics and Optics, St Petersburg, Russia

## Abstract

In linear optics, light fields do not mutually interact in a medium. However, they do mix when their field strength becomes comparable to electron binding energies in the so-called nonlinear optical regime. Such high fields are typically achieved with ultra-short laser pulses containing very broad frequency spectra where their amplitudes and phases are mutually coupled in a convolution process. Here, we describe a regime of nonlinear interactions without mixing of different frequencies. We demonstrate both in theory and experiment how frequency domain nonlinear optics overcomes the shortcomings arising from the convolution in conventional time domain interactions. We generate light fields with previously inaccessible properties by avoiding these uncontrolled couplings. Consequently, arbitrary phase functions are transferred linearly to other frequencies while preserving the general shape of the input spectrum. As a powerful application, we introduce deep UV phase control at 207 nm by using a conventional NIR pulse shaper.

## Introduction

While the control of nonlinear processes is typically very challenging, it is well known that many problems in engineering and computational physics can be routinely solved by addressing them in the Fourier domain. A paradigmatic example is the time dependent Schrödinger equation, a problem of first order in time and second order in space^1^. Applying a spatial Fourier transformation (FT) leads to an ordinary differential equation – a significant reduction of complexity.

We have adapted this universal approach by performing nonlinear optics^2^ in the frequency rather than in the time domain. We show that it is then possible to tailor the spectral properties of the nonlinearly generated field with unprecedented control. We introduce the term *Frequency domain Nonlinear Optics* (FNO)^3, 4^ to identify this approach and present an intuitive model to predict the resulting phenomenology.

**Figure 1:**
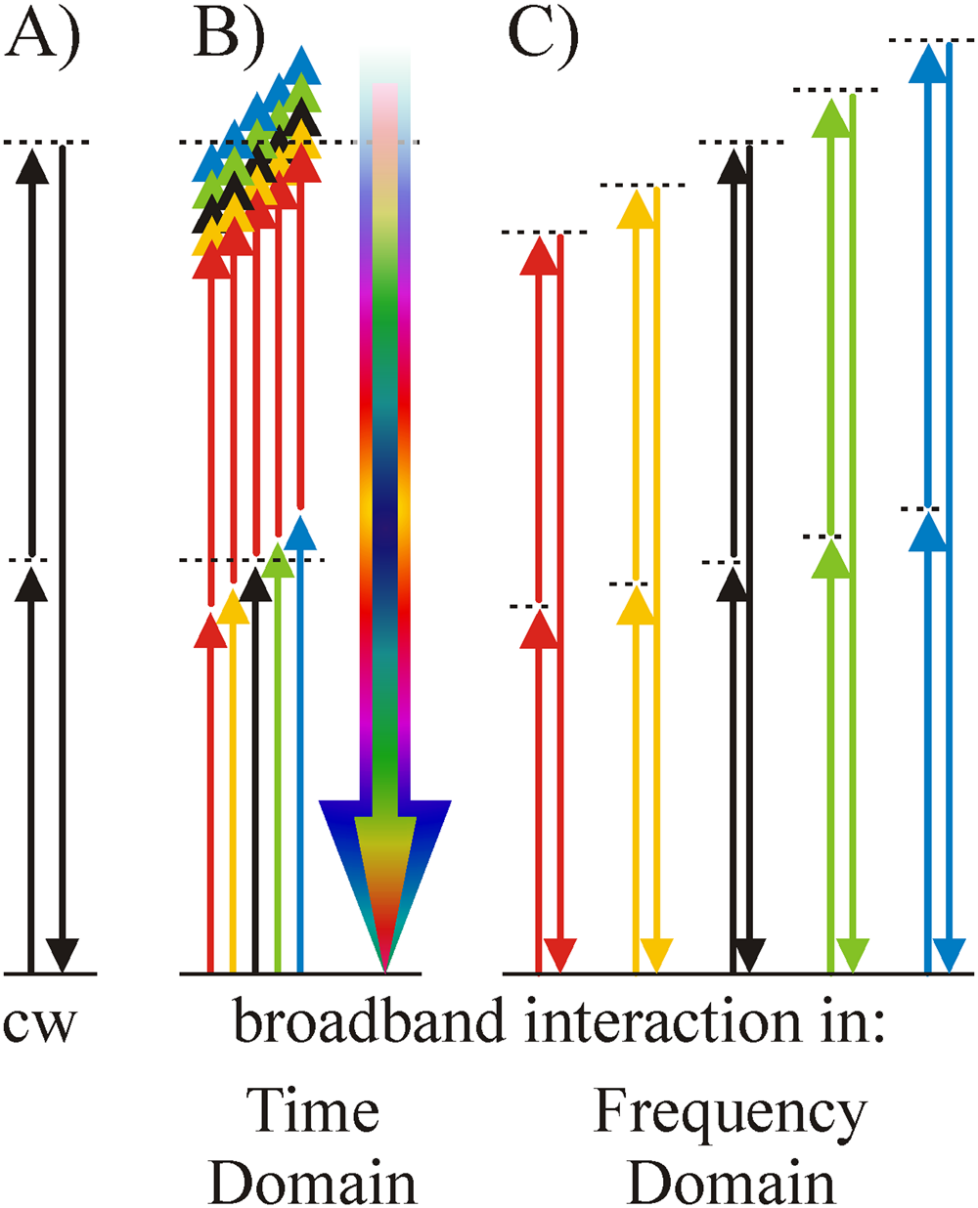
Energy diagrams for different scenarios of nonlinear interaction: (**A**) cw lasers, (**B**) broadband pulses in conventional time domain nonlinear optics (TNO) and (**C**) broadband pulses in frequency domain nonlinear optics (FNO). In TNO (**B**), all frequencies interact simultaneously leading to a mutual convolution of amplitudes and phases. In contrast, FNO (**C**) decouples the frequencies by performing a series of independent “quasi-cw” interactions prior to their recombination back into the time domain.

Employing FNO, we recently demonstrated broadband parametric amplification of energetic few-cycle laser pulses by avoiding the usual trade-off between optical gain and bandwidth^5^. This previous application realizes an idealized amplifier which does not change the pulse shape. The current work extends far beyond this important yet specific application: We demonstrate how the interaction of well-defined frequencies, as is shown in Fig. 1C, leads to the generation of new light fields which are inaccessible in conventional nonlinear optics (Fig. 1B). Furthermore, unlike traditional pulse shaping based on *linear manipulation* of light frequencies^6^, our approach enables *deterministic, nonlinear pulse shaping* in the frequency domain and it paves the way for new types of ultrafast spectroscopy. For the sake of clarity, we begin the discussion with second harmonic generation (SHG), since it was the first light induced optical nonlinearity - observed in 1961 by Franken and co-workers^7^. Nevertheless, our concept applies to other nonlinear effects such as *χ* ^(3)^ interactions as well as is described later and in the SI.

## Results

The basic physics underlying SHG can be presented in simple terms: Upon the excitation with an oscillating electric field at carrier frequency ω_0_, a nonlinear polarization wave is excited in the medium^8^. For a non-centrosymmetric material, the nonlinear polarization is proportional to the product of the input electric fields in the time domain: ${P}^{(2)}(t)={{\epsilon }}_{0}{\chi }^{(2)}{ {\mathcal E} }^{2}(t)$. Note, that we employ the complex notation for the electric field: ε (t) = Re[E(t)]. The fast oscillating component of this field acts as a source for electromagnetic radiation at twice the input frequency:

1 $${E}_{T}^{SH}(t)\propto {E}_{T}(t)\cdot {E}_{T}(t).$$

The subscript *T* identifies this type of interaction as the conventional *Time domain Nonlinear Optics* (TNO). The corresponding energy diagram for the case of a single frequency is shown in Fig. 1A. A mathematical FT leads to the spectral representation of Eq. (1):

2 $${E}_{T}^{SH}(\omega )\propto {E}_{T}(\omega )\,\ast \,{E}_{T}(\omega ).$$

**Figure 2:**
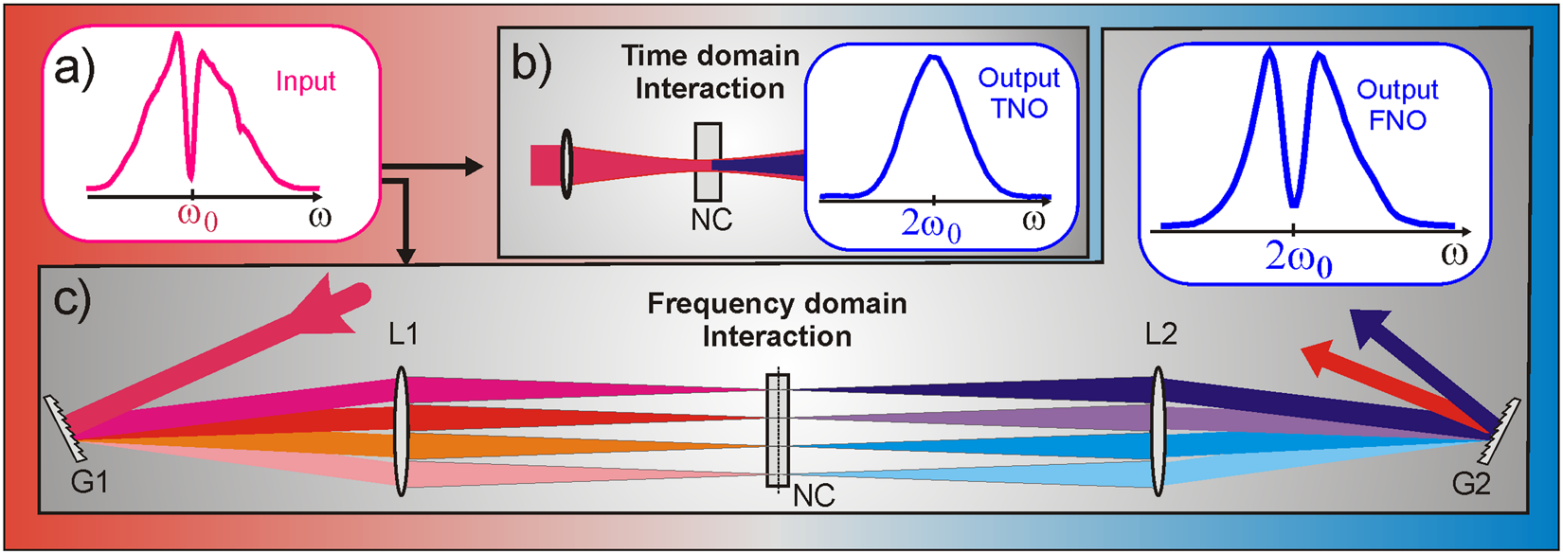
An input spectrum (**a**) is frequency doubled in a nonlinear crystal (NC) via (**b**) Time domain Nonlinear Optics (TNO) and via (**c**) Frequency domain Nonlinear Optics (FNO). (**b**) The experimental SH spectrum obtained by TNO (blue curve) shows a smooth function without the initial central hole. In FNO (**c**), the experimentally obtained SH spectrum at the output of the 4 f setup (blue curve) exhibits the same shape as the input spectrum.

The multiplication of E-fields in time changes to a convolution operation (*) in the frequency representation. While describing the nature of nonlinear interactions, at the same time this convolution imposes limitations in the case of ultrashort pulses consisting of many more than just one single frequency. Equation (2) expresses the spectral representation of TNO where *E*_*T*_(ω) contains the entire pulse spectrum, as depicted in Fig. 1B. The convolution operation mixes all spectral components of the input pulse, such that both, the SH amplitude and phase depend on all spectral components of the input pulse. In other words, an SHG process of broadband pulses unavoidably involves sum frequency mixing. As we will see later, this may cause substantial differences between an input spectrum (Fig. 2A) and its SH output spectrum (Fig. 2B).

The key aspect of FNO is the intrinsic ability to achieve a very narrow bandwidth for the nonlinear light-matter interaction even though the input and output pulses contain a broad bandwidth. The corresponding energy diagram for FNO is presented in Fig. 1C. Applying this scheme now enables pure SHG of broadband pulses without the omnipresent sum frequency mixing discussed for the TNO case. The narrow bandwidth in FNO is experimentally achieved by means of a *4-f* optical setup^9^,^10^ as described in Fig. 2C. The experimental setup visualizes the basic equation of FNO:

3 $${E}_{F}^{SH}(\omega )\propto {\int }_{-\infty }^{\infty }d\omega ^{\prime} \,({E}_{F}(\omega )\cdot \delta (\omega -\omega ^{\prime} ))\,\ast \,({E}_{F}(\omega )\cdot \delta (\omega -\omega ^{\prime} ))$$

The optical FT provides a reduced frequency content in each focal spot of the frequency plane (FP) which is expressed by the term ${E}_{F}(\omega )\cdot \delta (\omega -\omega ^{\prime} )$, where $\delta (\omega )\,\,$denotes the Dirac delta function. The subscript *F* refers to the optical interaction in the frequency domain. The integration over all frequency components (${\int }^{}d\omega ^{\prime}$) corresponds to the coherent recombination of all frequencies at the second grating G2 to form collimated output beams of the fundamental and SH (see Method section). Note that the convolution representing the nonlinear interaction is still present in Eq. (3). The basic nonlinear interaction in each focal point is of the same nature as in TNO. It applies, however, only to isolated, single frequencies. Thus, the mutual coupling between different colors is turned off. Evaluating Eq. (3) leads to (see *SI* section 1):

4 $${E}_{F}^{SH}(2\omega )\,\propto \,{E}_{F}(\omega )\cdot {E}_{F}(\omega )=|E(\omega ){|}^{2}{e}^{i2\varphi (\omega )},$$

where the spectral amplitude $|E(\omega )|$ and spectral phase $\varphi (\omega )\equiv Arg[E(\omega )]$ substantially differ from those obtained via TNO. The convolution of the E-fields in Eq. (2) is now changed to a simple multiplication in Eq. (4). This crucial difference is the foundation of FNO. We emphasize that Eq. (3) represents a generalized description of nonlinear wave mixing that also includes broadband interaction in time domain as a particular limit case, which is explained in section 2 of the SI. In the numerical simulations we replaced the delta function by a narrowband Gaussian distribution in accordance with the real experimental conditions.

### Amplitude transfer

We start the discussion of the far-reaching consequences of FNO by comparing the experimental power spectra obtained via Time domain SHG (TSH) and Frequency domain SHG (FSH) in Fig. 2. We first generate a transform limited (TL: flat spectral phase) 800 nm input pulse of 33 fs duration and add a hole in the centre of its power spectrum distribution (a). The pulse is then frequency doubled in a thin nonlinear crystal (NC). In both cases, we use a 150 µm thin BBO to minimize the role of phase-mismatch. In TNO (b), all frequencies interact simultaneously in a single focus and the experimental SH spectrum (blue curve) shows a smooth function without the initial central hole, a result of the convolution described in Eq. (2). Exploiting the setup drawn in Fig. 2c we generate an FSH pulse. The frequencies of an incident pulse disperse after the first grating G1 and their different propagation angles become parallel after the first lens L1. The lens again focuses each plane wave into a small focal spot next to its neighboring frequency. All frequency components, located side by side now form the frequency plane at one focal length behind the lens. This setup performs a continuous optical Fourier transformation from the time to the frequency domain. A symmetric setup subsequent to the frequency plane performs a second Fourier transformation back to the time domain. For the same input pulse and crystal used in the time domain experiment, we now obtain a completely different result: in the SH spectrum the central hole is preserved. Unlike in TSH, arbitrary amplitude transfers from the fundamental to the SH pulse are possible in FSH. This is a direct consequence of Eq. (4), which relates the SH power spectrum with its input counterpart via a phase independent relation that maintains a direct correspondence between the output and input frequencies: $I[2\omega ]={I}^{2}[\omega ]$. We also note that the generated SH output after the *4 f* setup exhibits excellent spatial properties, witnessed by the absence of spatial chirp and a round far field profile (see Fig. SI 5).

### Phase transfer

**Figure 3:**
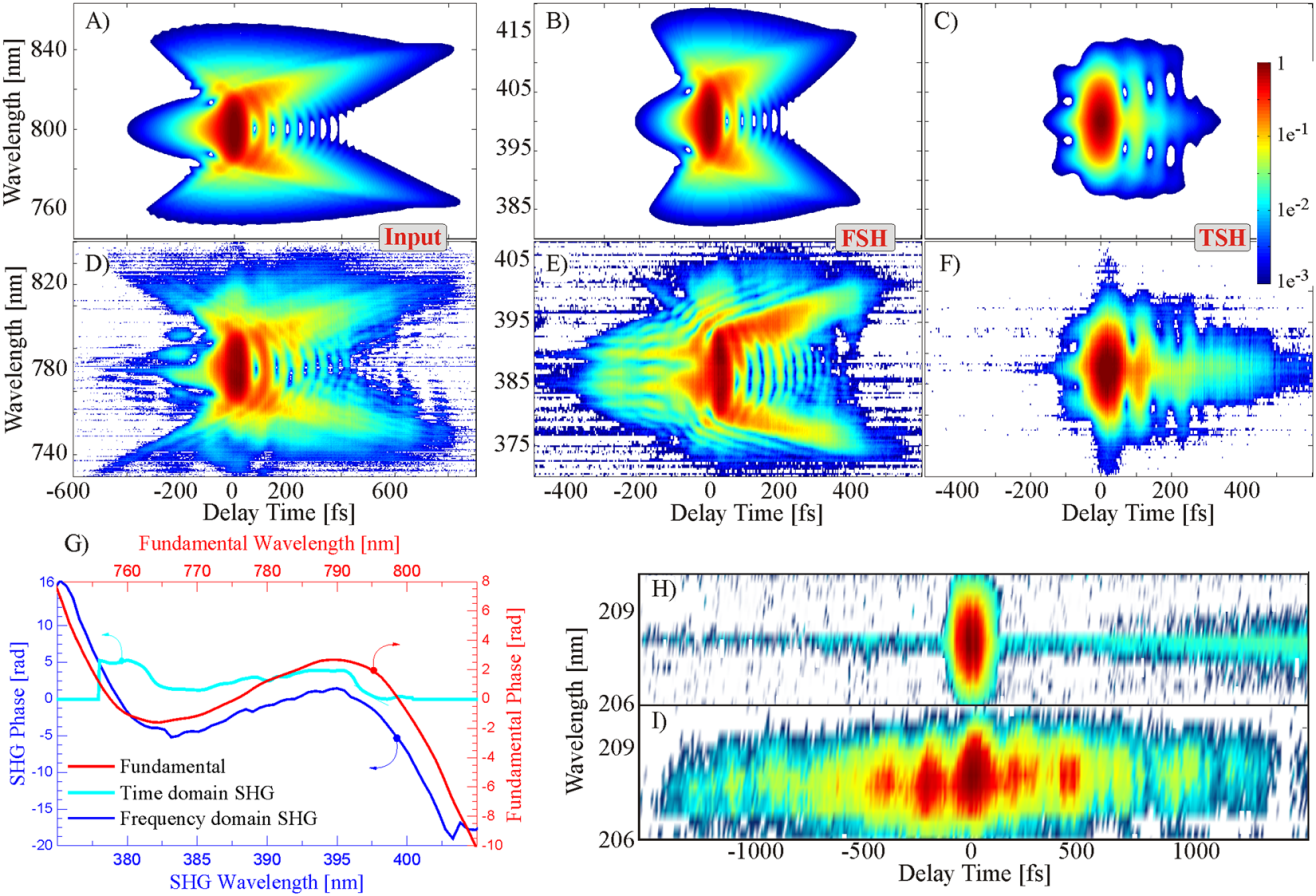
Theoretical (**A–C**) and experimental spectrograms at 800 nm (**D–F**) for FSH (**B,E**) and TSH (**C,F**) for pure phase shaping. In (**A**), a pure TOD of 150000 fs^3^ was added to a 33 fs TL fundamental pulse. In the experiment (**D**), additional higher order distortion terms are present. In FSH (**B**,**E**), the butterfly shape is nicely preserved while it is completely washed out in TSH (**C**,**F**). The experimentally retrieved phases are given in (**G**). The blue line showing the phase obtained through FSH nicely follows the shape of the fundamental (red line). The SH phase stroke is larger by a factor of 2 as expected from Eq. 5. This is evident from the different ranges for the left and right phase axes which refer to the second harmonic and the fundamental, respectively. The cyan line corresponding to TSH, on the contrary, exhibits a completely different behaviour. Thus, arbitrary phase functions can only be transferred via FSH. Even deep-UV shaping is possible. A TL pulse at 207 nm is shown in (**H**) and a pulse train achieved by applying a sine-phase function to the 830 nm input pulses in (**I**). Incidentally, these are the first FROG trace ever taken at such low wavelengths.

In TSH, only a linear chirp can be transferred from the input to the output phase (see SI section 3). The power spectrum, however, depends on the input chirp as we will discuss in the next section. The situation changes drastically when considering higher order phase functions. In that case, in addition to the power spectrum also the phase resulting from TSH is distorted. Therefore, the standard time domain approach fails to provide the means for an arbitrary spectral phase transfer between the input and the nonlinear output. On the other hand, we prove that FNO effectively maintains a linear input-output relation of the spectral phase in Eq. (4). Figure 3 provides an intuitive picture of the difference between TNO and FNO when comparing the spectrograms of a pulse exhibiting third order dispersion (TOD). Both numerical (a–c) and experimental (d–f) spectrograms, displaying the instantaneous pulse spectra for each delay point as a 2-d map, are shown. The spectrogram of a TL pulse has the shape of a symmetric balloon without distinct features. Applying a TOD of 150000 fs^3^, the typical shape of a butterfly with open wings illustrates how both spectral side bands are delayed with respect to the central frequency. The time domain approach (c) clearly results in a completely different spectrogram compared to the input (a). The butterfly closes its wings because at the trailing edge of the pulse both spectral side bands of the fundamental are annihilated to generate SH photons only around the central frequency $2{\omega }_{0}$. On the contrary, with FSH, the input (a) and output shape (b) are virtually the same. Note the logarithmic color bar, where green denotes the ~5% level. The calculations are in very good agreement with experiments (d-e).

We can extend this qualitative comparison to a quantitative analysis by retrieving the spectral phase from the measured spectrograms^11^. Figure 3(G) shows the retrieved fundamental (SH) phase in red (blue & cyan). Only the FSH phase (blue line) resembles the shape of the fundamental, thus the arbitrary phase is transferred linearly. We note that the output phase is doubled with respect to the input phase as predicted by Eq. (4) and that Δλ/λ_0_ is the same for both phase axes. In contrast, the cyan curve from TSH exhibits no correspondence to the fundamental phase. The experimental demonstration of SH pulse shaping through phase transfer in the FP has first been presented in Ref. 4 and extended to other pulse shapes in Ref. 12. As a further, powerful application of FNO we introduce arbitrary phase control of the 4^th^ harmonic, at a wavelength as short as 207 nm, see Fig. 3(H). Direct phase control was hitherto demonstrated down to 270 nm, limited by the availability of optical pulse shaping crystals^13^. We surpassed this limit by imaging the first FP onto a subsequent one with an additional BBO doubling crystal, to reach the 4^th^ harmonic. Due to phase matching limitations in BBO, the shortest possible wavelength to be accessed via direct frequency quadrupling of a TiSa laser is 205 nm. To leave some room for phase matching tuning, we aimed at a UV wavelength centered around 207 nm. Noteworthy, this was done without additional gratings in between both FPs, i.e. between both subsequent doubling stages. Omitting the recombination grating for the 415 nm beam automatically provides the angular dispersion required for the FP of the second doubling stage. For the sake of simplicity, the 4^th^ harmonic output was recombined using the same grating as at 800 nm, but now in its 4^th^ diffraction order. Alternatively, a different grating with higher groove density could be used to recombine the corresponding harmonics of the fundamental beam. We like to note that the pulse train in Fig. 3(I) at 207 nm is achieved through sin-phase shaping applied to the TiSa fundamental prior to the multi-pass amplifier. More details are found in section 8 of the SI. We like to stress that while direct phase transfer is also possible in TNO with certain limitations, it has so far only been demonstrated in cross correlation types of interaction^14, 15^. In FNO unrestricted phase transfer can occur with a single input beam.

In general, FNO can provide a direct relation between complex input and output fields, which are connected by a variable *n* that depends on the nonlinear process. For instance, in the case of SHG *n* = 2 in Eq. 4, for third harmonic generation *n* = 3 (see SI section 6), while for parametric amplification *n* equals 1 meaning that the phase and spectral shape remain unchanged. If we neglect absorption and phase matching constraints, Eq. 4 can be generalized in the case of instantaneous nonlinear effects as:

5 $${E}_{F}^{n}(n\omega )\,\propto \,|E(\omega ){|}^{n}{e}^{in\varphi (\omega )}.$$

As Eq. 5 shows, in FNO, the output amplitude follows the power of *n*, while the phase depends linearly on *n*.

### Amplitude and phase coupling

**Figure 4:**
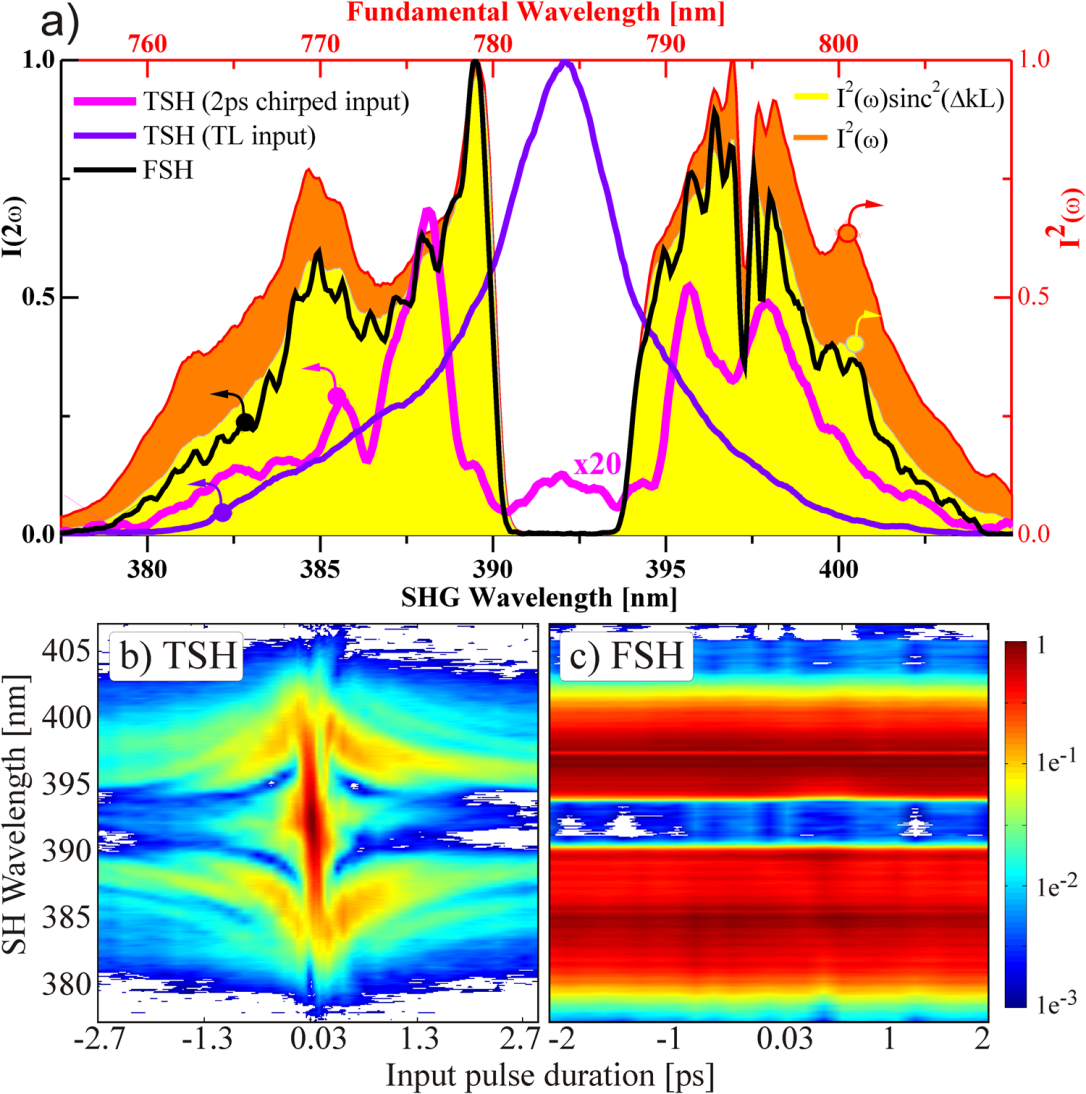
Investigating amplitude and phase coupling as a function of chirp. The amplitude of the fundamental was shaped according to the filled orange curve in (**a**), which shows the square of the input intensity. TSH (**c**) exhibits a strong dependence of the spectral amplitude as a function of input chirp. Because this amplitude-phase coupling is absent in FSH (**c**), all SH spectra remain constant, like the black line in (**a**). All spectral features transfer directly to the SH.

We now demonstrate how input pulses with both, amplitude and phase variations give rise to different outputs for the two schemes. As mentioned before, even for a Gaussian input pulse with a pure linear chirp, the power spectrum obtained through TSH depends on the input spectral phase, i.e. a chirped input generates less SH. We now discuss how this dependence affects the SH process, and how amplitudes and phases are decoupled by performing the nonlinear interaction in the frequency domain. To this end, and for the sake of clarity, we consider a pulse featuring a distinct hole in the spectrum, shown in Fig. 4a as the filled orange curve. Its SH is first generated in the time domain, and the resulting power spectrum is recorded for linear chirps varying from –17600 fs^2^ to 17600 fs^2^. This stretches a TL pulse from 33 fs to as much as 2.7 ps. The results in Fig. 4b show significant changes of the SH power spectrum as a function of chirp. Again, for a TL pulse (flat phase), the central hole is completely absent (blue line in Fig. 4a). The central hole only develops as the pulse gets temporally stretched (magenta line: input stretched to 2 ps). Even in this extreme case where different frequencies arrive at different times, the mutual coupling is only reduced but not completely suppressed. Furthermore, the SHG efficiency changes significantly, a consequence of the chirp-dependent pulse intensity. The situation is radically different in the FSH result depicted in (c). The power spectrum (black line in Fig. 4a) obtained for the same input conditions as in (b) does not change irrespective of the input phase. Eq. (4), however, predicts a perfect match of the orange curve in (a) with the black plot since: $I(2\omega )\propto {I}^{2}(\omega )$. The observed discrepancy can be easily explained. It arises from the limited phase-matching bandwidth provided by the birefringence of the 150 µm thick BBO (see SI section 4). Accounting for this macroscopic effect leads to the prediction plotted as the yellow filled curve.

## Discussion and Outlook

Since it is difficult to analyze feature sharpness in TSH where the SH spectrum strongly depends on the input shape and phase, we investigate the time span required to develop the spectral hole introduced in the input beam (orange curve of Fig. 4a). The TL duration of the fundamental is about 33 fs FWHM. The missing central part corresponds to a TL duration of about 360 fs FWHM (zero-zero duration ~1 ps). The magenta line in (a) for a chirped input of ~2 ps still shows features at the central wavelength indicating that a much longer temporal stretching is required to minimize the mutual coupling of frequencies in TNO. Based on this estimation we conclude that amplitude and phase coupling can only be turned off efficiently in FSH. The clear one-to-one match of the experimental FSH spectrum with the ideal phase matched fundamental one in Fig. 4a, together with the linear phase transfer of ﻿arbitrary ﻿﻿﻿functions in Fig. 3G support the interpretation of a “linearization” of the nonlinear process. For coherent processes and in particular for nonlinear optics the phases mostly define the total output. The linearization arises through the spectral separation and coherent recombination of individual phases afterwards.

The loss of bandwidth appears to be even more pronounced in TSH than in FSH (as is seen by comparing the black line to the blue and magenta ones in Fig. 4a). In order to compensate the loss of bandwidth due to phase mismatch, the standard time domain approach offers the choices of using thinner crystals with less efficiency, or elaborated achromatic phase matching schemes^16^. While a variation of the latter schemes can also be realized in FSH, it offers additional implementations based on, e.g., the use of periodically poled materials^17^ or the use of the separation Ansatz^18^ with multiple thick crystals to efficiently double an octave of frequencies. Although in TNO frequency conversion of chirped pulses may be improved according to a more complex adiabatic scheme^19^, the problem of recompression afterwards still remains - which is not the case in FNO.

From a practical point of view we like to mention that working in a FP reduces the intensity in the interaction region depending on the geometrical optical arrangement^18^. While this is a clear advantage for power scaling it sets limits for the minimum input energy required. Considering the use of nonlinear crystals with high nonlinearity like periodically poled materials^17^, an input energy of about 1µJ is required to reach an intensity of 1 GW/cm^2^ in a typical 4 f setup.

Another application of our scheme could be the coherent combination of single-cycle pulses with their single-cycle SH to generate multi-octave sub-cycle light fields^20^ or to widen the current frequency bands used for metrology^21^.

We also envision a modified type of 2D spectroscopy^22^ by placing a sample in the FP and utilize the spread colors as a narrow band pump in each focal spot. Next, all spots can be simultaneously probed by a time delayed fs pulse.

Moreover, in addition to advancing current spectroscopy methods, FNO could inspire novel schemes. One idea is to interfere the SH outputs of TNO and FNO and to record the phase difference as a function of input intensity. While the FNO phase will remain constant, the TNO phase should vary as a function of intensity. Thus, nonlinear phase shifts could be detected even at extreme wavelengths. This approach could be further expanded from a non-resonant to a resonant two-photon excitation. One might also consider the possibility of interfering higher order harmonics generated through perturbative nonlinear optical approaches via FNO with those produced through strong field effects. This, in turn, will allow us to gain insight into the mechanism of below-bandgap high order harmonics in solids^23^.

Furthermore, the so “linearized nonlinear optics” could also extend the functionality of integrated optical devices^24^ and logical operations^25, 26^.

Ultimately, a large number of nonlinear effects discovered during the last half century can be revisited in the frequency domain. However, different results can be achieved due to the elimination of the mutual couplings of frequencies upon nonlinear interactions, together with the possibility of direct phase and amplitude transfer to other wavelengths.

In summary, frequency domain nonlinear optics elegantly merges the simplicity of linear optics with the power of conventional nonlinear optics by decoupling frequencies, amplitudes and phases.

## Methods

### Laser system

The measurements were carried out at the advanced Laser Light Source (ALLS) with 1 mJ, 33 fs pulses at 780 nm wavelength from a Ti:Sa amplifier operating at 500 Hz. The beam diameter was 4 mm FWHM carrying 500 µJ of pulse energy. Amplitude and phase-shaping was carried out with an acousto-optic programmable dispersive filter (AOPDF, DAZZLER). The results in Fig. 4 showing the chirp dependence were obtained by changing the grating separation in the compressor. Spectral measurements were carried out with an integration sphere fiber-coupled to an imaging spectrometer. Pulse characterization was performed with an all reflective transient grating – frequency resolved optical gating (TG-FROG) apparatus. Beam separation of the three beams was carried out with a transmission mask consisting of three holes that enabled wavelength and polarization independent operation. The same device can measure the fundamental, SH, and fourth harmonic pulses regardless of their polarization. Unlike SHG-FROG, it has no time ambiguity and generates intuitive spectrograms for quadratic and cubic phase functions, thus facilitating phase retrieval and data interpretation.

Details concerning the deep-UV shaping setup are given in section SI8.

### *4* *f setup*

A 4 f setup comprised of reflection gratings (600 lines/mm) and lenses (f = 300 mm), as shown in Fig. 2c, was used in the experiment. Additionally, a half wave plate was inserted prior to L1 to rotate the initially perpendicular polarization of the fundamental by 90°. This sole modification is not shown in Fig. 2c for the sake of clarity. Since a 150 µm thick type I BBO is used for doubling, the SH polarization is perpendicular to the fundamental. The half wave plate between the gratings ensures perpendicular polarization of the SH beam on the exit grating. This allows angle tuning of the BBO phase matching along the spectrally dispersed axis. Additionally, one can achieve more efficient grating operation at the SH wavelength if the polarization is perpendicular to the grating grooves. The long focal lengths of L1 & L2 lead to a rather low intensity (around 1 GW/cm2) in the FP. This, in combination with the thin BBO crystal (150 µm) yielded an SHG efficiency in the FP of about 5%. The setup was not optimized for SHG efficiency (due to the low intensity in the FP) and the thin BBO was chosen because thicker crystals showed pronounced phase matching deficiencies that make comparison between FSH and TSH difficult.

The long focal lengths also eased modifications and allowed access to the FP. The efficiency should increase with shorter focal lengths, higher pulse energy or thicker crystals. The overall SH efficiency might be further improved by choosing a grating blazed for 400 nm with twice the groove size of the input grating satisfying the condition: $\sin (\alpha ){\lambda }_{800nm}/g=\,\sin (\alpha ){\lambda }_{400nm}/2g$. In this way, it would not be possible to recombine the fundamental if the grating is designed for the SH. However, we chose the same grating at the exit side for convenience and because it can recombine either the fundamental (at angle α) or the SH beam (at angle 2α). The SH beam recombines through 2^nd^ order diffraction while the fundamental beam recombines via 1st order diffraction. The SH beam exhibits no spatial chirp and the far field measurement shows a perfectly symmetric focal spot containing all spectral components. We found, however, that fundamental and SH beams recombine optimally for slightly different incident angles on the second grating G2. We note that this might be related to the angular acceptance bandwidth of BBO. The bandwidth and corresponding pulse duration in the focal spot of the FP depend on the geometric optical properties of the setup:

6 $$\Delta {\lambda }_{foc}=\frac{{\lambda }_{c}g}{\pi \,{d}_{in}}2\,\mathrm{ln}(2)\sqrt{1-{(\frac{{\lambda }_{c}}{2g})}^{2}}$$

7 $$\Delta {t}_{foc}=\frac{{\lambda }_{c}{d}_{in}}{\,gc\,}\,{[1-{(\frac{{\lambda }_{c}}{2g})}^{2}]}^{-1/2}$$

Being *Δλ*_*foc*_ the bandwidth in the focal spot of the FP, *λ*_*c*_ the center wavelength, *g* the groove density, *d*_*in*_ the input beam diameter, and *Δt*_*foc*_ the effective pulse duration in the FP. In typical experimental arrangements, the focal bandwidth *Δλ*_*foc*_ in the FP is > 100 times smaller than the input pulse spectrum. The finest spectral feature transferred via FSH is given by the spectral resolution of the 4 *f* setup, equal to n *Δλ*_*foc*_.

### Data availability statement

The datasets generated during and/or analysed during the current study are available from the corresponding author on reasonable request.

## Electronic supplementary material

Supplementary Info
