# Supplementary material for: Decoupling Frequencies, Amplitudes and Phases in Nonlinear Optics
Source: Sci Rep. 2017 Aug 11;7:7861. doi: 10.1038/s41598-017-07510-3 (PMC5554166; doi:10.1038/s41598-017-07510-3)
Supplement: Supplementary file 1 — Supplementary Info [file 41598_2017_7510_MOESM1_ESM.pdf]

Decoupling Frequencies, amplitudes and phases in nonlinear optics: supplementary material

Bruno E. Schmidt[1,2]*, Philippe Lassonde[2], Guilmot Ernotte[2], Matteo Clerici[3], Roberto Morandotti[2], Heide Ibrahim[2] and François Légaré[2]†

[1]few-cycle Inc., 2890 Rue de Beaurivage, Montreal, H1L 5W5, Qc, Canada
[2]INRS-EMT, 1650 Blvd. Lionel Boulet, Varennes, J3X 1S2, Qc, Canada
[3]University of Glasgow, School of Engineering, G12 8QQ, Glasgow, UK
Correspondence to: *schmidt@few-cycle.com, †legare@emt.inrs.ca

This file includes:
Supplementary Text
Figs. S1 to S7

S1 - Nonlinear interaction in the Frequency domain.

This section is dedicated to the derivation of Eq. (4) describing the nonlinear interaction in the Frequency domain. In the Fourier plane (FP), the initial spectrum is spatially separated such that only a "single" frequency *w'* is present in each focal spot. Therefore the spectrum in one focal spot can be expressed as the product of the initial spectrum and a delta distribution: $E(w)\cdot\delta(w-w')$. This reduced bandwidth interacts nonlinearly in the BBO crystal. The nonlinear interaction is represented by a convolution in the frequency representation as in Eq. (2) of the main paper. Finally, the last grating recombines all frequency together, as represented by an integral over all *w'*. Thus, the nonlinear interaction in a *4f* setup can be expressed as:

$$E_F^{SH}(w) \sim \int_{-\infty}^{\infty} dw' \, \big(E(w)\cdot\delta(w-w')\big) * \big(E(w)\cdot\delta(w-w')\big). \qquad \text{(S 1)}$$

After performing the multiplication with the Dirac delta, only the convolution of two deltas remains. Note that $E(w')$ is not a function of w any more:

$$E_F^{SH}(w) \sim \int_{-\infty}^{\infty} dw' \, E(w')\cdot E(w')\cdot\delta(w-w') * \delta(w-w'). \qquad \text{(SI 2)}$$

According to the convolution properties of the Dirac delta $\delta(x-a)*\delta(x-b)=\delta(x-a-b)$, this equation can be written as follows:

$$E_F^{SH}(w) \sim \int_{-\infty}^{\infty} dw' \, E(w')\cdot E(w')\cdot\delta(w-2w'). \qquad \text{(SI 3)}$$

$$E_F^{SH}(w) \sim \int_{-\infty}^{\infty} dw' \, E(w')\cdot E(w')\cdot\delta\left(-2\left(w'-\frac{w}{2}\right)\right). \qquad \text{(SI 4)}$$

With $\delta\left(\frac{x}{b}\right) = |b|\delta(x)$

$$E_F^{SH}(w) \sim \int_{-\infty}^{\infty} dw' \, E(w')\cdot E(w')\cdot\delta\left(w'-\frac{w}{2}\right). \qquad \text{(SI 5)}$$

$$E_F^{SH}(w) \sim \int_{-\infty}^{\infty} dw' \, E\left(\frac{w}{2}\right)\cdot E\left(\frac{w}{2}\right)\cdot\delta\left(w'-\frac{w}{2}\right). \qquad \text{(SI 6)}$$

This regular convolution integral is evaluated using the properties of the Dirac delta and leads to:

$$E_F^{SH}(w) \sim E\left(\frac{w}{2}\right)E\left(\frac{w}{2}\right) \qquad \text{(SI 7)}$$

Therefore, the spectrum of the second harmonic in the time domain is the square of the initial spectrum. The *w/2* term (representing the fundamental frequency) accommodates the fact that the frequency axis needs to be expanded by a factor of 2. This will have the effect to broaden the function by a factor of 2 and to move the center of the function from the fundamental to its second harmonic (as expected for SHG). Broadening by a factor of 2 holds for $E_F^{SH}(w)$ while on the other hand, $I_F^{SH}(w)$ will be broader by $\sqrt{2}$ as expected for a SH process in the absence of phase matching restrictions. In Eq. (4) of the main paper we substituted $\omega = w/2$.

S2 - Approximation using δ function

One important point that needs to be addressed is the validity of the "single" frequency assumption in the FP, on which the introduction of the Kronecker delta function is actually based. We will discuss two approaches to justify this assumption.

First, we will examine our picture from a geometrical optics point of view. The 4f setup not only performs a *temporal* Fourier transformation from the time to the frequency domain, but it also carries out a *spatial* Fourier transformation. The latter merely reflects the main purpose of a focusing optic, i.e. it indeed focuses a parallel beam into a focal spot, or it transforms a point source into a collimated beam, respectively. This point source can be seen as a delta function whose Fourier transform yields a constant function, the plane wave. This idealized picture disregards the effect of finite optic sizes. If the 4f setup is illuminated with a polychromatic plane wave, each frequency leaves the grating under a different diffraction angle while still being a plane wave. The focusing optic performs a spatial Fourier transformation of each plane wave to generate a delta function. An inclined, plane wave incident onto a lens (i.e. different pulse frequencies) corresponds to a function with a linear phase slope with respect to the spatial coordinate. This linear phase slope causes a displacement in the Fourier transform plane, thus all frequencies get spatially separated.

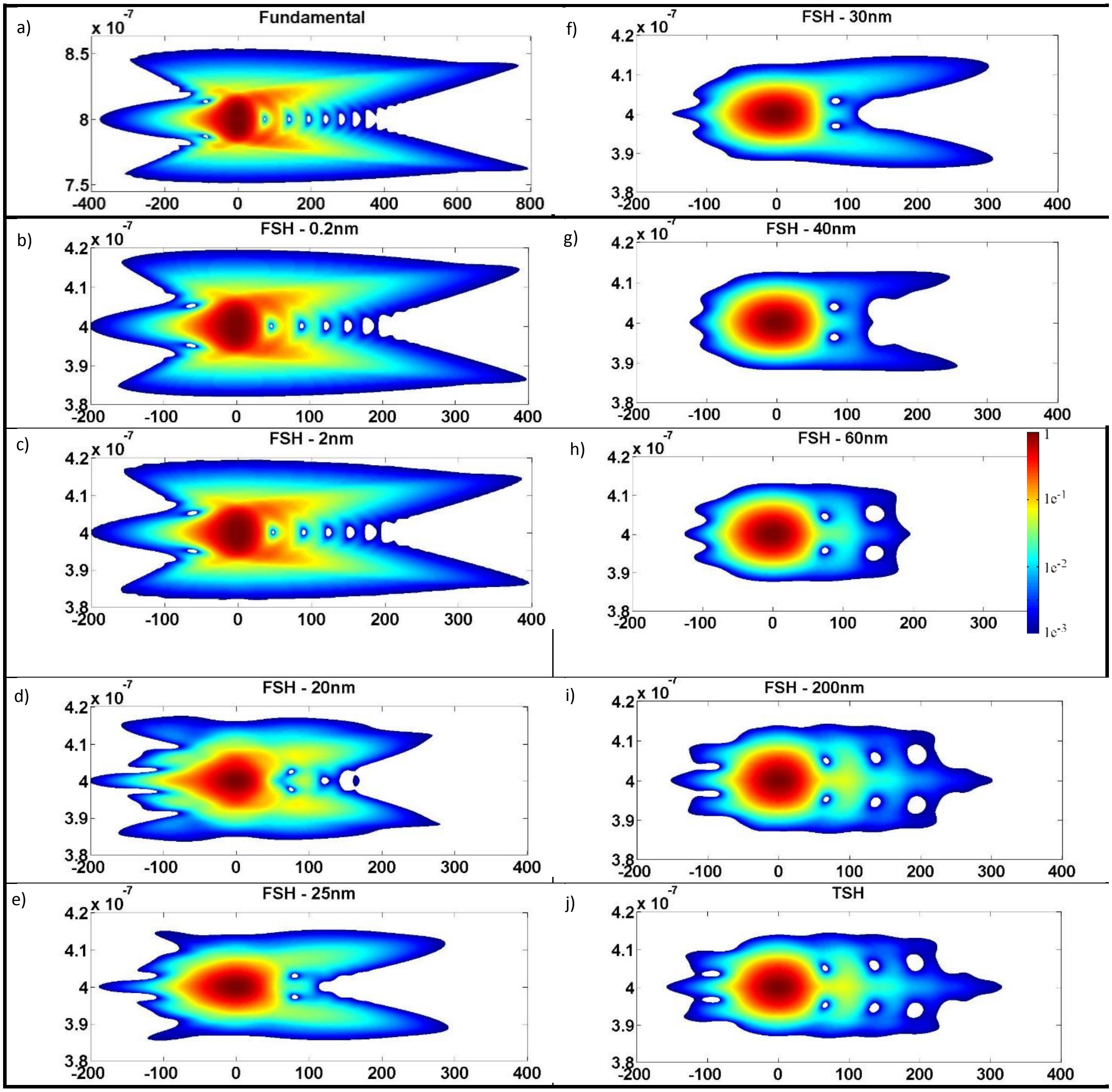

Figure SI 1: Spectrograms illustrating the influence of varying resolution of the 4f setup in the Fourier plane (b-i). (a) shows the fundamental pulse with a FWHM of 28nm and (j) the time domain SHG. The experimental resolution of the 4f setup was 0.1nm. As this resolution is decreased, one can follow the transition from FNO to TNO. Note the logarithmic scale.

Second, to demonstrate the validity of the delta function approximation, we perform numerical simulations according to Eq. (SI 1) assuming a variable focal spot size in the FP. We point out that the numerical simulation describes the generation process, not the propagation of ultrashort pulses. Note that in the numerical code we replace the delta function by a Gaussian to resemble the real experimental conditions of the focal spot in the FP. This is a realistic assumption and takes into account the influence of finite optics sizes. Changing the focal spot size is equivalent to modifying the effective spectral bandwidth for the nonlinear interaction. The FWHM bandwidth of a TL 33fs pulse corresponds to 28nm. In the experiment, the FWHM bandwidth in each focus of the FP spot was roughly 0.2nm, i.e. about 300 times smaller than the pulse bandwidth. Figure SI 1 shows the evolution from FNO to TNO as the effective bandwidth in the 4f setup approaches the FWHM bandwidth of the pulse (i.e., as the spectral resolution decreases).

For a low enough resolution of the 4f setup, the result of FNO equals TNO (compare i) and j)) , i.e. FSH at the lowest resolution and TSH). This means that Eq. (3) of the main paper can be thought as a more general description for nonlinear optical interactions, ***where the universally used TNO approach is effectively a limit case of FNO when the spectral resolution is largely reduced***.

S3 - Transfer of other phase functions

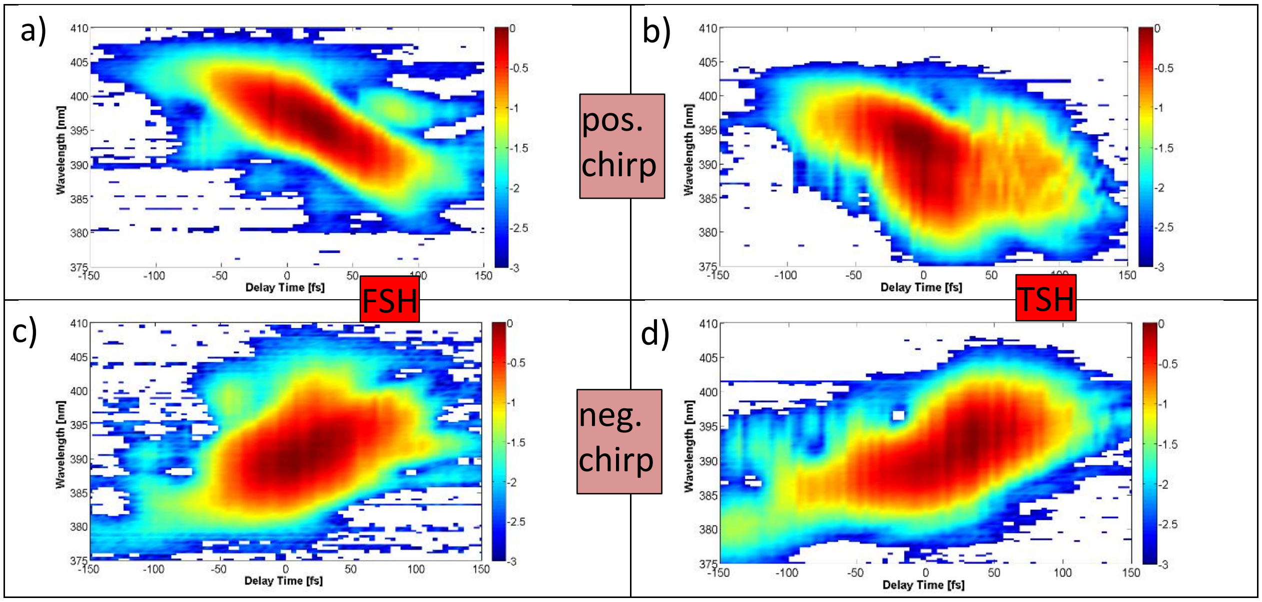

Figure SI 2: Transfer of liner chirp in FSH and TSH for both positive and negative signs, (a, b) fundamental, (c, d) SH.

As mentioned in the main paper, (only) second order phases can be transferred via TNO, an important fact that is confirmed by the experimental spectrograms in Fig. SI 2 (b) & (d). The different sign of the fundamental input chirp (not shown) is transferred to the SH. The same is true for FNO.

For completeness, we also show the spectrograms for an opposite TOD sign as compared to the results of Fig. 3 in the main paper. Due to experimental limitations, the quality of phase shaping is reduced. The reason is that we aligned the acoustic wave settings inside the AOPDF such that it enables the maximum amount of chirp in one direction (+ 150 000 fs^3).

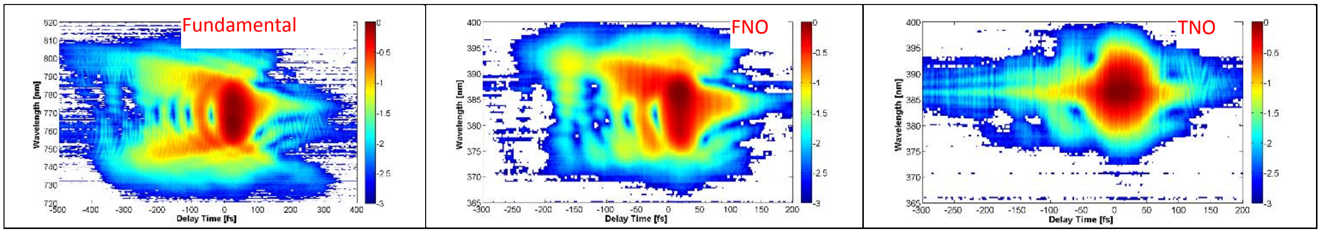

Figure SI 3: Phase shaping with opposite sign of TOD

S4 - Role of phase matching

In a macroscopic medium, there are two factors, besides absorption, which limit the useful bandwidth. The first, acting at the microscopic scale, is related to the photon order of the process involved. A SH process requires two fundamental photons for the emission of a photon at twice the photon energy. This quadratic dependence of the output power spectral density on the input intensity leads to a spectral narrowing which is illustrated in Fig. SI 4. The blue curve shows the square of the input spectrum, represented in red. The amount of narrowing depends on the shape of the function involved. The second limiting factor is related to the fact that, in the case of an extended medium, macroscopic propagation effects will occur. The most important one is phase matching which is required for a constructive superposition of coherent waves. The maximum power spectrum that is phase-matched in SHG (based on birefringent phase matching) is given by: $sinc^2(\Delta kL)$, plotted as the green curve in Fig. SI 4. L is half the crystal thickness and Δk is the wave vector mismatch between all three interacting waves.

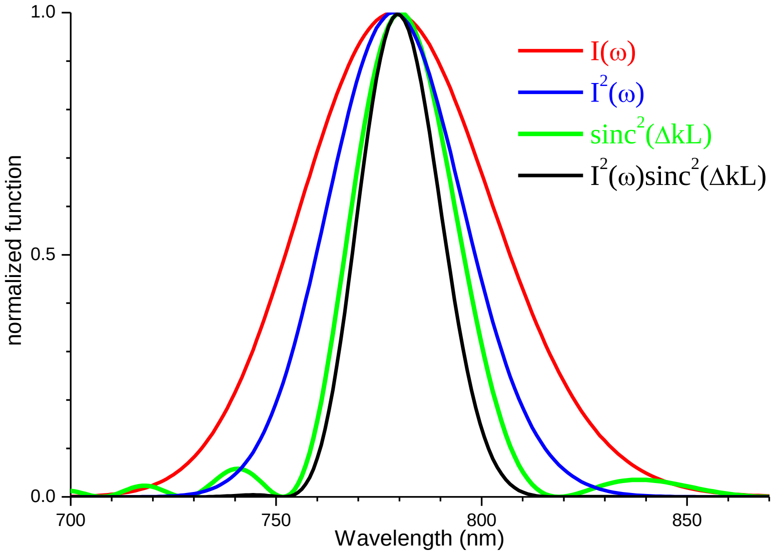

Figure SI 4: Incident fundamental bandwidth (red), ideal SH output bandwidth (blue) and effective phase-matched bandwidth (black)

The final phase matched bandwidth for a SH process is the multiplication of both effects: $I^2(w)sinc^2(\Delta kL)$, plotted here as the black curve. We point out that this restriction applies to both TNO and FNO. FNO, however, offers the chance to double a higher relative bandwidth than TNO if a spectral shape different than Gaussian is chosen as the input function.

S5 - 4f setup output properties

As mentioned in the main paper, the output properties of the 4f setup at the SH wavelength are well emphasized by the absence of spatial chirp matched to good focusing properties. An image of the focal spot is shown in Fig. SI 5.

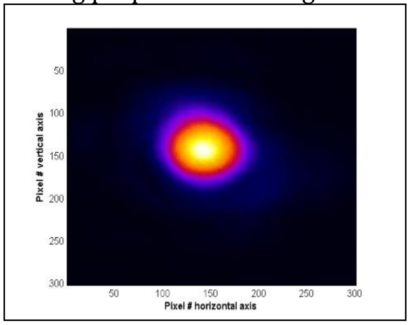

Figure SI 5: Focal spot of FSH output

Another indication of good spatial quality is the TL pulse duration measured with the TG-FROG. Here, a plate with three spatially separated holes is used to provide the three beams for the four wave mixing process. Only if the spectral contend across the entire beam is spatially uniform, it is possible to measure TL pulse durations. The otherwise reduced bandwidth leads to longer pulse durations.

S6 - Third harmonic generation (THG)

The time domain representation of third order nonlinearities can be described by [1] (page 10):

$$E^{TH}(t) \propto E(t) \cdot E(t) \cdot E(t) \qquad \text{SI (8)}$$

which leads to the frequency representation

$$E^{TH}(w) \propto E(w) * E(w) * E(w) \qquad \text{SI (9)}$$

Analog with FSH, Fourier domain THG (FTH) can be performed and described analytically following the approach presented in SI (1):

$$E_F^{TH}(w) \sim \int_{-\infty}^{\infty} dw' \ \left(E_F(w) \cdot \delta(w-w')\right) * \left(E_F(w) \cdot \delta(w-w')\right) * \left(E_F(w) \cdot \delta(w-w')\right) \qquad \text{SI(10)}$$

$$E_F^{TH}(w) \sim \int_{-\infty}^{\infty} dw' \ E_F(w') \cdot E_F(w') \cdot E_F(w') \cdot \left(\delta(w-w') * \delta(w-w') * \delta(w-w')\right) \qquad \text{SI (11)}$$

$$E_F^{TH}(w) \sim \int_{-\infty}^{\infty} dw' \, E_F^3(w') \cdot (\delta(w-2w') * \delta(w-w')) \qquad \text{SI (12)}$$

$$E_F^{TH}(w) \sim \int_{-\infty}^{\infty} dw' \, E_F^3(w') \cdot \delta(w-3w') \qquad \text{SI (13)}$$

$$E_F^{TH}(w) \sim \int_{-\infty}^{\infty} dw' \, E_F^3(w') \cdot \delta\left(-2\left(w' - \frac{w}{3}\right)\right) \qquad \text{SI (14)}$$

$$E_F^{TH}(w) \sim E_F^3\left(\frac{w}{3}\right) \qquad \text{SI (15)}$$

$$E_F^{TH}(3w) \sim E_F^3(w) \qquad \text{SI (16)}$$

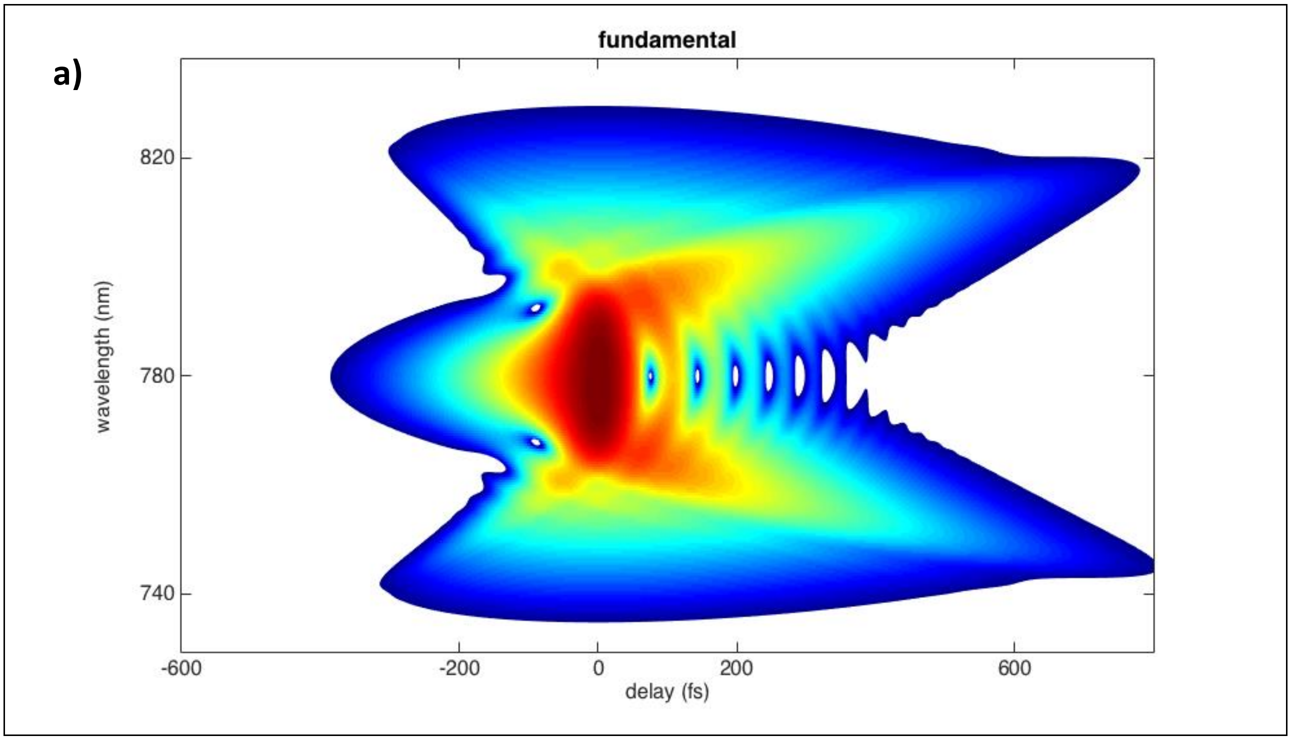

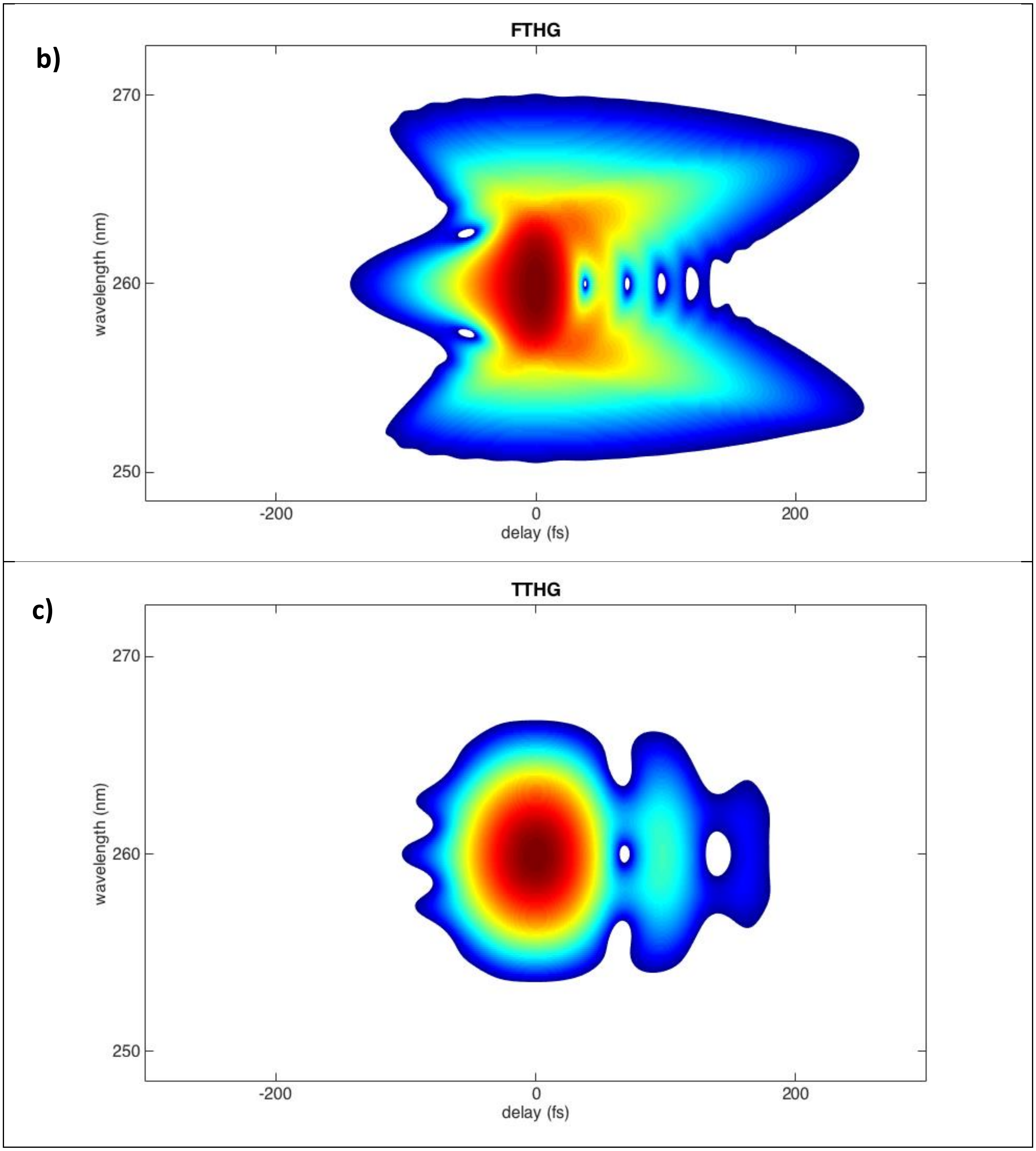

Fig. SI 6: Extending frequency domain nonlinear optics to third order nonlinearities. Shown in (a) is the same fundamental input pulse as in Fig. 3(a) of the main paper carrying a large TOD. (b) and (c) show the numerical results for third harmonic generation in the frequency and time domain, respectively.

S7 - Second harmonic efficiency

We would like to point out that there is no intrinsic trade-off with regard to efficiency. However, the current FNO set-up requires a reduced intensity on the nonlinear medium, which results in a lower nonlinear response. In other words, the reason for the lower intensity is simply the enlarged focal spot size (i.e. a long line in the Fourier plane) and the orders of magnitude longer pulse durations.
However, this is not necessary a limitation, as our approach carries an intrinsic advantage with regard to the ability of doubling high energy pulses.
Furthermore, in our experiment the intensity (around 1GW/cm^2) was low because we used long focal lengths (300mm). Typical intensities for BBO can go beyond 100GW/cm^2. This particular geometry allowed us to have easy access to the

Fourier plane and try different configurations, also giving us the additional ability of inserting other optical elements, such as the mentioned wave plate. As far as efficiency of low energy pulses is concerned, there is a number of changes that could be made, including:
1) decreasing the focal length
2) using multiple, thicker crystals like in the case of FOPA (*Nat. Commun.* **5,** 3643 (2014))
3) in fact, the separated frequencies in the Fourier plane are perfectly suited for periodically poled crystals with a corresponding fan-out structure. These devices actually work in the intensity range used in our experiment.
In a follow up deep-UV generation experiment, the SH efficiency in a modified setup (described in the next section) reached 30%. The total setup efficiency including the transmission of standard gratings is about 17% from pump in the FP to SH output after the 4f setup.

S8 – Deep UV pulse shaping

The path to generate the 4th harmonic of a TiSa laser is conceptually straight forward. It is based on successive doubling of the second harmonic in a subsequent FP. The bigger challenge is to deal with the limited choice of optics as well as with the poor sensitivity of current diagnostic devices at deep UV wavelengths.
Due to phase matching limitations in BBO, the shortest possible wavelength to be accessed via direct frequency quadrupling of a TiSa laser is 205nm. To leave some room for phase matching tuning, we aimed at a UV wavelength centred around 207nm. Thus, the TiSa fundamental had to be shifted from 790nm in the previous experiment to 830nm. At this centre wavelength, the pulse duration increased to about 80fs due to a narrower available spectrum out of the TiSa amplifier, with 360µJ of energy incident on the first grating. While using the same gratings as before, we redesigned the first FP based on 200mm lenses and a 300µm thick BBO. This enabled to increase the intensity and hence the doubling efficiency to 30% in the first FP. The second grating of the first 4f setup was removed. Instead, another 100mm lens was placed 100mm after the position of the original output grating. In this way, the angular dispersion of the first FP is transferred to the next one without need for other gratings in between. To remove the fundamental after the first FP, a BG39 absorption filter was used, whose transmission at 400nm was only 50%. The 100mm lens generated a second FP in which a 100µm thick type I BBO (theta = 78°) was placed for fourth harmonic generation (FHG). Subsequently, a 100mm lens and the same 600 line grating (aluminium coated) as at the 800nm input was used to recombine the FH output beam in the 4th diffraction order. While using the 4th diffraction order is very convenient, however, it is also quite lossy. We estimated the efficiency to be 5-10%. As in previous experiments, we precisely aligned the incident angle until the spatial chirp (in both direction) became negligible. Measuring the focal spot at 207nm was not possible due to the low CCD sensitivity this wavelength. In particular, the remaining 400nm stray light prevented such a measurement.
All lenses of the two FPs were AR coated except the last one, used to recombine the FH beam. The overall energy efficiency for converting the fundamental to the FH including all losses was 0.02%, corresponding to 70nJ pulse energy. After all propagation and transport optics we measured an energy of 25nJ in front of the TG-FROG. The TG-FROG is identical to the one used before, except all silver mirrors were changed to aluminium.
Replacing the BG39 filter by a dichroic beam splitter, using AR coated lenses at 200nm and changing the last grating to a 2400 line grating should yield a total efficiency of 0.5%. Additionally, multiple BBO crystals could be used for more efficient and, simultaneously, more broadband frequency conversion.
Our most striking result, the generation of a fs pulse train centred at 207nm wavelength is presented in fig. 3(I) of the main paper. This was achieved by applying a sine-phase mask with the DAZZLER after the TiSa oscillator. Without sine-phase, a TL pulse of 100fs was measured. To further confirm the phase transfer capabilities we also carried out TOD shaping which is presented in Fig. SI 7. Comparing the red shaded curve (fundamental spectrum) with the blue shaded curve (FH spectrum) shows a significant difference of the spectral shape. This change of shape can be fully explained by phase matching effects as explained in section S6. The phase matching at 207nm is extremely narrowband. Consequently, the relative bandwidth $({}^{\Delta\lambda}/_{\lambda})$ of the FH pulse is 2.3 times smaller compared to the fundamental pulse. In other words, multiplying the shaded red curve with a 2.3 times more narrow sinc function will necessarily lead to a symmetric output spectrum, only due to phase matching constraints. More important for practical applications, however, is the successful transfer of a higher order phase function, which is proven by the features of the retrieved phases shown as the solid lines. The red curve corresponds to the fundamental while the blue curve represents the FH phase. Its height of phase stroke corresponding to about 13 radians is not 4 times the one of the fundamental (8 radians) as we would expect from Eq. (5) in the main paper. This result is strongly consistent, however, with the loss of phase matching bandwidth, since the height of phase stroke at is about 2.4 times less as expected when phase matching effects are neglected.

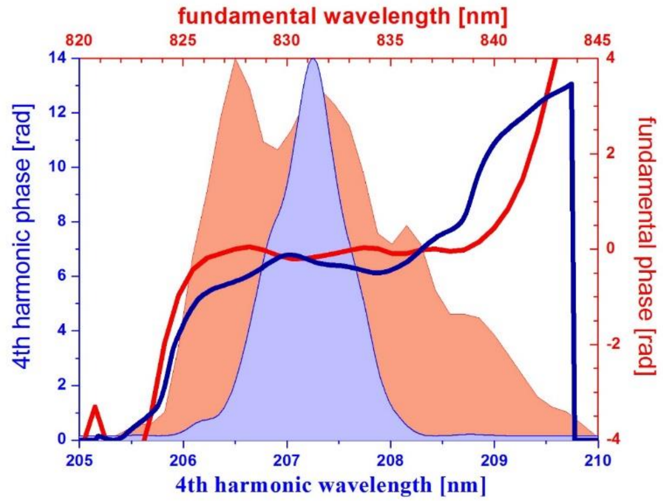

Fig. SI 7: TOD phase transfer from 830nm to 207nm. The red (blue) curves correspond to the retrieved spectral amplitude and phase of the fundamental (fourth harmonic) pulses.
